# 1-(Adamantan-1-yl)-3-(4-meth­oxy­phenyl)prop-2-en-1-one^1^

**DOI:** 10.1107/S1600536812032400

**Published:** 2012-07-25

**Authors:** Thy M. Nguyen, Frank R. Fronczek, Steven F. Watkins

**Affiliations:** a Department of Chemistry, Louisiana State University, Baton Rouge, LA 70803-1804, USA

## Abstract

The title mol­ecule, C_20_H_24_O_2_, is a chalconoid derivative in which the keto–enone group is slightly distorted from planarity; the O=C—C=C torsion angle is 12.24 (13)°.

## Related literature

For the role of the keto-enone group in chalconoid chemistry, see: Homan *et al.* (1997 ▶). Many chalconoid derivatives are bioactive agents with anti­flammatory, anti­tumor, anti­viral, gastroprotective and/or mutagenic activity, see: Ravishankar *et al.* (2003 ▶); Sathiya Moorthi *et al.* (2005 ▶); Patil *et al.* (2006 ▶). Some adamantane derivatives have shown anti­viral activity, especially against influenza and herpes viruses, see: Mullica *et al.* (1999 ▶). For the synthesis, see: Kazlov *et al.* (1995 ▶).
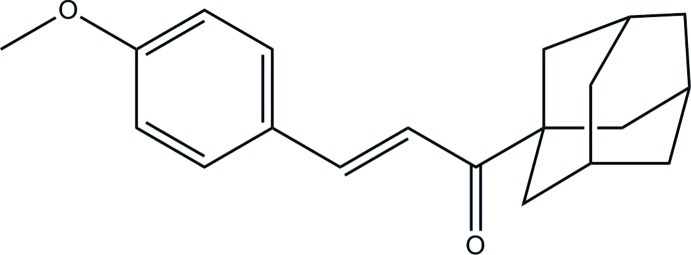


## Experimental

### 

#### Crystal data

C_20_H_24_O_2_
*M* *_r_* = 296.39
Monoclinic, 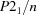

*a* = 6.4648 (1) Å
*b* = 16.4712 (3) Å
*c* = 14.6134 (4) Å
β = 92.612 (1)°
*V* = 1554.46 (6) Å^3^
*Z* = 4
Mo *K*α radiation
μ = 0.08 mm^−1^
*T* = 90 K
0.35 × 0.28 × 0.24 mm

#### Data collection

Nonius KappaCCD diffractometer
Absorption correction: multi-scan (*SCALEPACK*; Otwinowski & Minor, 1997 ▶) *T*_min_ = 0.973, *T*_max_ = 0.981
11653 measured reflections
6165 independent reflections
4860 reflections with *I* > 2σ(*I*)
*R*_int_ = 0.026

#### Refinement

*R*[*F* ^2^ > 2σ(*F* ^2^)] = 0.045
*wR*(*F* ^2^) = 0.123
*S* = 0.99
6165 reflections
200 parameters
H-atom parameters constrained
Δρ_max_ = 0.47 e Å^−3^
Δρ_min_ = −0.29 e Å^−3^

### 

Data collection: *COLLECT* (Nonius, 2000 ▶); cell refinement: *SCALEPACK* (Otwinowski & Minor, 1997 ▶); data reduction: *DENZO* (Otwinowski & Minor, 1997 ▶) and *SCALEPACK*; program(s) used to solve structure: *SHELXS97* (Sheldrick, 2008 ▶); program(s) used to refine structure: *SHELXL97* (Sheldrick, 2008 ▶); molecular graphics: *ORTEP-3 for Windows* (Farrugia, 1997 ▶); software used to prepare material for publication: *WinGX* (Farrugia, 1999 ▶).

## Supplementary Material

Crystal structure: contains datablock(s) global, I. DOI: 10.1107/S1600536812032400/ds2206sup1.cif

Structure factors: contains datablock(s) I. DOI: 10.1107/S1600536812032400/ds2206Isup2.hkl

Supplementary material file. DOI: 10.1107/S1600536812032400/ds2206Isup3.cml

Additional supplementary materials:  crystallographic information; 3D view; checkCIF report

## Figures and Tables

**Fig. 1.**
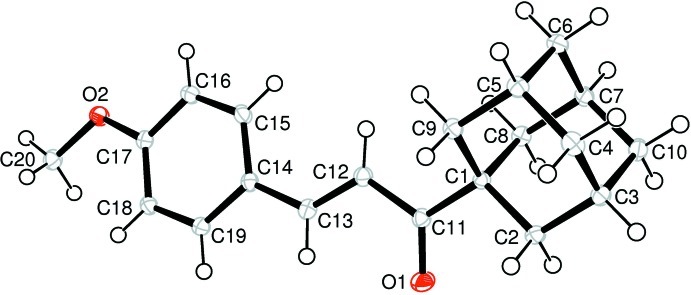
View of (I) (50% probability displacement ellipsoids)

## supplementary crystallographic information

### Comment

Many chalconoid derivatives are bioactive agents with antiflammatory, antitumor, antiviral, gastroprotective and/or mutagenic activity (Ravishankar *et al.*, 2003; Sathiya Moorthi *et al.*, 2005; Patil *et al.*, 2006). Title compound I is a chalconoid derivative with an adamantyl group in place of an aromatic system. Some adamantane derivatives have shown antiviral activity, specially against influenza and herpes viruses (Mullica *et al.*, 1999).

The keto-enone group in particular plays a significant role in chalconoid chemistry since it is susceptible to a number of transformations (Homan *et al.*, 1997). In I, this group is not planar: the O=C—C=C torsion is 12.24 (13)°. All other bond lengths and bond angles are within expected norms.

### Experimental

The title compound was synthesized by Dr. Gabriel Garcia following the procedure of Kazlov *et al.* (1995). A suitable crystal was obtained by recrystallization from ethanol.

### Refinement

All H atoms were placed in calculated positions, guided by difference maps, with C—H bond distances constrained to the range 0.95–1.00 Å, and *U*_iso_=1.2*U*_eq_ (1.5 for the methyl group), thereafter refined as riding. A torsional parameter was also refined for the methyl group.

### Crystal data

**Table:**

C_20_H_24_O_2_	*F*(000) = 640
*M**_r_* = 296.39	*D*_x_ = 1.266 Mg m^−^^3^
Monoclinic, *P*2_1_/*n*	Mo *K*α radiation, λ = 0.71073 Å
Hall symbol: -P 2yn	Cell parameters from 5846 reflections
*a* = 6.4648 (1) Å	θ = 2.6–33.7°
*b* = 16.4712 (3) Å	µ = 0.08 mm^−^^1^
*c* = 14.6134 (4) Å	*T* = 90 K
β = 92.612 (1)°	Prism, colorless
*V* = 1554.46 (6) Å^3^	0.35 × 0.28 × 0.24 mm
*Z* = 4	

### Data collection

**Table:**

Nonius KappaCCD diffractometer	6165 independent reflections
Radiation source: sealed tube	4860 reflections with *I* > 2σ(*I*)
Graphite monochromator	*R*_int_ = 0.026
Detector resolution: 9 pixels mm^-1^	θ_max_ = 33.7°, θ_min_ = 2.8°
CCD rotation images, thick slices scans	*h* = −10→10
Absorption correction: multi-scan (*SCALEPACK*; Otwinowski & Minor, 1997)	*k* = −25→24
*T*_min_ = 0.973, *T*_max_ = 0.981	*l* = −22→22
11653 measured reflections	

### Refinement

**Table:**

Refinement on *F*^2^	Primary atom site location: structure-invariant direct methods
Least-squares matrix: full	Secondary atom site location: difference Fourier map
*R*[*F*^2^ > 2σ(*F*^2^)] = 0.045	Hydrogen site location: inferred from neighbouring sites
*wR*(*F*^2^) = 0.123	H-atom parameters constrained
*S* = 0.99	*w* = 1/[σ^2^(*F*_o_^2^) + (0.0625*P*)^2^ + 0.485*P*] where *P* = (*F*_o_^2^ + 2*F*_c_^2^)/3
6165 reflections	(Δ/σ)_max_ < 0.001
200 parameters	Δρ_max_ = 0.47 e Å^−^^3^
0 restraints	Δρ_min_ = −0.29 e Å^−^^3^
0 constraints	

### Special details

**Table:**

Geometry. All e.s.d.'s (except the e.s.d. in the dihedral angle between two l.s. planes) are estimated using the full covariance matrix. The cell e.s.d.'s are taken into account individually in the estimation of e.s.d.'s in distances, angles and torsion angles; correlations between e.s.d.'s in cell parameters are only used when they are defined by crystal symmetry. An approximate (isotropic) treatment of cell e.s.d.'s is used for estimating e.s.d.'s involving l.s. planes.

### Fractional atomic coordinates and isotropic or equivalent isotropic displacement parameters (Å^2^)

**Table:**

	*x*	*y*	*z*	*U*_iso_*/*U*_eq_	
C1	1.17484 (12)	0.49689 (5)	0.77117 (6)	0.01203 (14)	
C2	1.39267 (13)	0.50311 (5)	0.81790 (6)	0.01455 (15)	
H2A	1.4965	0.5105	0.7709	0.017*	
H2B	1.3994	0.5509	0.859	0.017*	
C3	1.44258 (12)	0.42587 (5)	0.87340 (6)	0.01529 (16)	
H3	1.585	0.4303	0.9027	0.018*	
C4	1.43213 (13)	0.35203 (5)	0.80922 (7)	0.01668 (16)	
H4A	1.4671	0.3021	0.8444	0.02*	
H4B	1.534	0.3584	0.7611	0.02*	
C5	1.21310 (13)	0.34455 (5)	0.76470 (6)	0.01470 (15)	
H5	1.2062	0.296	0.7236	0.018*	
C6	1.05468 (13)	0.33524 (5)	0.83940 (6)	0.01542 (15)	
H6A	0.9136	0.3304	0.8107	0.019*	
H6B	1.0848	0.2853	0.8753	0.019*	
C7	1.06570 (13)	0.40947 (5)	0.90296 (6)	0.01396 (15)	
H7	0.9626	0.4032	0.9515	0.017*	
C8	1.01594 (12)	0.48667 (5)	0.84674 (6)	0.01350 (15)	
H8A	1.0212	0.5346	0.8876	0.016*	
H8B	0.8744	0.4827	0.8182	0.016*	
C9	1.16385 (13)	0.42133 (5)	0.70825 (6)	0.01387 (15)	
H9A	1.0236	0.4168	0.6786	0.017*	
H9B	1.2645	0.427	0.6596	0.017*	
C10	1.28430 (13)	0.41594 (6)	0.94802 (6)	0.01611 (16)	
H10A	1.3163	0.3664	0.9844	0.019*	
H10B	1.2919	0.4632	0.9899	0.019*	
C11	1.12481 (13)	0.57368 (5)	0.71656 (6)	0.01414 (15)	
C12	0.93169 (13)	0.57598 (5)	0.65717 (6)	0.01481 (15)	
H12	0.8298	0.5348	0.6616	0.018*	
C13	0.90363 (13)	0.63728 (5)	0.59715 (6)	0.01471 (15)	
H13	1.0173	0.6735	0.5934	0.018*	
C14	0.72429 (13)	0.65630 (5)	0.53694 (6)	0.01366 (15)	
C15	0.53920 (13)	0.61095 (5)	0.53374 (6)	0.01574 (16)	
H15	0.5296	0.5635	0.5703	0.019*	
C16	0.37129 (13)	0.63471 (5)	0.47793 (6)	0.01635 (16)	
H16	0.2482	0.6032	0.4758	0.02*	
C17	0.38227 (13)	0.70527 (5)	0.42450 (6)	0.01399 (15)	
C18	0.56503 (13)	0.75027 (5)	0.42526 (6)	0.01432 (15)	
H18	0.5747	0.7973	0.3881	0.017*	
C19	0.73332 (13)	0.72522 (5)	0.48129 (6)	0.01446 (15)	
H19	0.8579	0.7559	0.4817	0.017*	
C20	0.21429 (14)	0.79562 (5)	0.31728 (6)	0.01815 (17)	
H20A	0.2512	0.8432	0.3549	0.027*	
H20B	0.0786	0.8043	0.2861	0.027*	
H20C	0.3187	0.7875	0.2716	0.027*	
O1	1.23918 (11)	0.63302 (4)	0.72203 (5)	0.02329 (16)	
O2	0.20598 (10)	0.72512 (4)	0.37467 (5)	0.01787 (14)	

### Atomic displacement parameters (Å^2^)

**Table:**

	*U*^11^	*U*^22^	*U*^33^	*U*^12^	*U*^13^	*U*^23^
C1	0.0108 (3)	0.0126 (3)	0.0126 (3)	0.0000 (2)	0.0000 (2)	−0.0001 (3)
C2	0.0118 (3)	0.0153 (4)	0.0165 (4)	−0.0015 (3)	−0.0007 (3)	0.0012 (3)
C3	0.0114 (3)	0.0175 (4)	0.0168 (4)	0.0001 (3)	−0.0013 (3)	0.0023 (3)
C4	0.0146 (3)	0.0163 (4)	0.0193 (4)	0.0042 (3)	0.0033 (3)	0.0022 (3)
C5	0.0169 (3)	0.0122 (3)	0.0151 (4)	0.0011 (3)	0.0019 (3)	−0.0010 (3)
C6	0.0159 (3)	0.0142 (4)	0.0162 (4)	−0.0018 (3)	0.0019 (3)	0.0005 (3)
C7	0.0133 (3)	0.0164 (4)	0.0123 (4)	−0.0003 (3)	0.0025 (3)	0.0003 (3)
C8	0.0119 (3)	0.0151 (4)	0.0137 (4)	0.0017 (3)	0.0020 (3)	−0.0011 (3)
C9	0.0154 (3)	0.0138 (4)	0.0125 (4)	0.0006 (3)	0.0016 (3)	−0.0011 (3)
C10	0.0163 (3)	0.0187 (4)	0.0132 (4)	0.0002 (3)	−0.0010 (3)	0.0008 (3)
C11	0.0144 (3)	0.0136 (4)	0.0143 (4)	0.0008 (3)	−0.0005 (3)	0.0002 (3)
C12	0.0141 (3)	0.0148 (4)	0.0154 (4)	0.0006 (3)	−0.0007 (3)	0.0006 (3)
C13	0.0157 (3)	0.0139 (4)	0.0144 (4)	0.0017 (3)	−0.0005 (3)	−0.0010 (3)
C14	0.0150 (3)	0.0134 (3)	0.0126 (4)	0.0015 (3)	0.0005 (3)	−0.0002 (3)
C15	0.0179 (4)	0.0143 (4)	0.0150 (4)	0.0000 (3)	0.0008 (3)	0.0026 (3)
C16	0.0153 (3)	0.0168 (4)	0.0169 (4)	−0.0018 (3)	0.0008 (3)	0.0020 (3)
C17	0.0139 (3)	0.0151 (4)	0.0129 (3)	0.0012 (3)	0.0000 (3)	−0.0002 (3)
C18	0.0160 (3)	0.0135 (3)	0.0135 (4)	0.0005 (3)	0.0008 (3)	0.0013 (3)
C19	0.0147 (3)	0.0140 (4)	0.0147 (4)	−0.0005 (3)	0.0004 (3)	0.0004 (3)
C20	0.0208 (4)	0.0154 (4)	0.0179 (4)	0.0016 (3)	−0.0032 (3)	0.0016 (3)
O1	0.0247 (3)	0.0153 (3)	0.0289 (4)	−0.0052 (2)	−0.0089 (3)	0.0040 (3)
O2	0.0146 (3)	0.0193 (3)	0.0194 (3)	−0.0003 (2)	−0.0026 (2)	0.0047 (2)

### Geometric parameters (Å, º)

**Table:**

C1—C11	1.5223 (12)	C9—H9B	0.99
C1—C2	1.5402 (11)	C10—H10A	0.99
C1—C9	1.5471 (12)	C10—H10B	0.99
C1—C8	1.5513 (12)	C11—O1	1.2260 (11)
C2—C3	1.5351 (12)	C11—C12	1.4882 (12)
C2—H2A	0.99	C12—C13	1.3443 (12)
C2—H2B	0.99	C12—H12	0.95
C3—C4	1.5355 (13)	C13—C14	1.4572 (12)
C3—C10	1.5378 (13)	C13—H13	0.95
C3—H3	1	C14—C19	1.3992 (12)
C4—C5	1.5361 (12)	C14—C15	1.4095 (12)
C4—H4A	0.99	C15—C16	1.3842 (12)
C4—H4B	0.99	C15—H15	0.95
C5—C9	1.5354 (12)	C16—C17	1.4037 (12)
C5—C6	1.5382 (12)	C16—H16	0.95
C5—H5	1	C17—O2	1.3639 (10)
C6—C7	1.5351 (12)	C17—C18	1.3943 (12)
C6—H6A	0.99	C18—C19	1.3939 (12)
C6—H6B	0.99	C18—H18	0.95
C7—C10	1.5354 (12)	C19—H19	0.95
C7—C8	1.5398 (12)	C20—O2	1.4348 (11)
C7—H7	1	C20—H20A	0.98
C8—H8A	0.99	C20—H20B	0.98
C8—H8B	0.99	C20—H20C	0.98
C9—H9A	0.99		
			
C11—C1—C2	110.20 (7)	H8A—C8—H8B	108.2
C11—C1—C9	110.73 (7)	C5—C9—C1	109.91 (7)
C2—C1—C9	109.47 (7)	C5—C9—H9A	109.7
C11—C1—C8	109.37 (7)	C1—C9—H9A	109.7
C2—C1—C8	108.26 (7)	C5—C9—H9B	109.7
C9—C1—C8	108.77 (6)	C1—C9—H9B	109.7
C3—C2—C1	110.19 (7)	H9A—C9—H9B	108.2
C3—C2—H2A	109.6	C7—C10—C3	109.42 (7)
C1—C2—H2A	109.6	C7—C10—H10A	109.8
C3—C2—H2B	109.6	C3—C10—H10A	109.8
C1—C2—H2B	109.6	C7—C10—H10B	109.8
H2A—C2—H2B	108.1	C3—C10—H10B	109.8
C2—C3—C4	109.35 (7)	H10A—C10—H10B	108.2
C2—C3—C10	109.40 (7)	O1—C11—C12	120.34 (8)
C4—C3—C10	109.68 (7)	O1—C11—C1	121.00 (7)
C2—C3—H3	109.5	C12—C11—C1	118.65 (7)
C4—C3—H3	109.5	C13—C12—C11	118.97 (8)
C10—C3—H3	109.5	C13—C12—H12	120.5
C3—C4—C5	109.65 (7)	C11—C12—H12	120.5
C3—C4—H4A	109.7	C12—C13—C14	129.45 (8)
C5—C4—H4A	109.7	C12—C13—H13	115.3
C3—C4—H4B	109.7	C14—C13—H13	115.3
C5—C4—H4B	109.7	C19—C14—C15	117.94 (7)
H4A—C4—H4B	108.2	C19—C14—C13	118.18 (7)
C9—C5—C4	109.03 (7)	C15—C14—C13	123.84 (8)
C9—C5—C6	109.56 (7)	C16—C15—C14	120.76 (8)
C4—C5—C6	109.76 (7)	C16—C15—H15	119.6
C9—C5—H5	109.5	C14—C15—H15	119.6
C4—C5—H5	109.5	C15—C16—C17	120.24 (8)
C6—C5—H5	109.5	C15—C16—H16	119.9
C7—C6—C5	109.64 (7)	C17—C16—H16	119.9
C7—C6—H6A	109.7	O2—C17—C18	124.33 (8)
C5—C6—H6A	109.7	O2—C17—C16	115.67 (7)
C7—C6—H6B	109.7	C18—C17—C16	120.00 (8)
C5—C6—H6B	109.7	C19—C18—C17	119.06 (8)
H6A—C6—H6B	108.2	C19—C18—H18	120.5
C10—C7—C6	109.34 (7)	C17—C18—H18	120.5
C10—C7—C8	109.71 (7)	C18—C19—C14	121.97 (8)
C6—C7—C8	109.39 (7)	C18—C19—H19	119
C10—C7—H7	109.5	C14—C19—H19	119
C6—C7—H7	109.5	O2—C20—H20A	109.5
C8—C7—H7	109.5	O2—C20—H20B	109.5
C7—C8—C1	109.90 (6)	H20A—C20—H20B	109.5
C7—C8—H8A	109.7	O2—C20—H20C	109.5
C1—C8—H8A	109.7	H20A—C20—H20C	109.5
C7—C8—H8B	109.7	H20B—C20—H20C	109.5
C1—C8—H8B	109.7	C17—O2—C20	116.96 (7)
			
C11—C1—C2—C3	179.68 (7)	C2—C3—C10—C7	59.86 (9)
C9—C1—C2—C3	−58.31 (9)	C4—C3—C10—C7	−60.08 (9)
C8—C1—C2—C3	60.12 (9)	C2—C1—C11—O1	−8.16 (12)
C1—C2—C3—C4	59.33 (9)	C9—C1—C11—O1	−129.42 (9)
C1—C2—C3—C10	−60.81 (9)	C8—C1—C11—O1	110.73 (9)
C2—C3—C4—C5	−60.59 (9)	C2—C1—C11—C12	172.81 (7)
C10—C3—C4—C5	59.37 (9)	C9—C1—C11—C12	51.55 (10)
C3—C4—C5—C9	60.99 (9)	C8—C1—C11—C12	−68.30 (9)
C3—C4—C5—C6	−59.03 (9)	O1—C11—C12—C13	12.24 (13)
C9—C5—C6—C7	−60.23 (9)	C1—C11—C12—C13	−168.72 (8)
C4—C5—C6—C7	59.47 (9)	C11—C12—C13—C14	−175.10 (8)
C5—C6—C7—C10	−60.07 (9)	C12—C13—C14—C19	179.04 (9)
C5—C6—C7—C8	60.10 (9)	C12—C13—C14—C15	1.35 (15)
C10—C7—C8—C1	59.94 (9)	C19—C14—C15—C16	−0.75 (13)
C6—C7—C8—C1	−60.01 (8)	C13—C14—C15—C16	176.95 (8)
C11—C1—C8—C7	−179.62 (6)	C14—C15—C16—C17	−0.90 (14)
C2—C1—C8—C7	−59.53 (8)	C15—C16—C17—O2	−177.53 (8)
C9—C1—C8—C7	59.34 (8)	C15—C16—C17—C18	2.08 (14)
C4—C5—C9—C1	−60.05 (9)	O2—C17—C18—C19	178.00 (8)
C6—C5—C9—C1	60.10 (9)	C16—C17—C18—C19	−1.57 (13)
C11—C1—C9—C5	−179.57 (7)	C17—C18—C19—C14	−0.10 (13)
C2—C1—C9—C5	58.74 (9)	C15—C14—C19—C18	1.25 (13)
C8—C1—C9—C5	−59.36 (8)	C13—C14—C19—C18	−176.58 (8)
C6—C7—C10—C3	60.33 (9)	C18—C17—O2—C20	2.20 (13)
C8—C7—C10—C3	−59.64 (9)	C16—C17—O2—C20	−178.21 (8)
